# Jasmonic Acid Effect on the Fatty Acid and Terpenoid Indole Alkaloid Accumulation in Cell Suspension Cultures of *Catharanthus roseus*

**DOI:** 10.3390/molecules190710242

**Published:** 2014-07-15

**Authors:** Guitele Dalia Goldhaber-Pasillas, Natali Rianika Mustafa, Robert Verpoorte

**Affiliations:** Natural Products Laboratory, Institute of Biology Leiden, Leiden University, Sylvius Laboratoria, Sylviusweg 72, 2300 RA, Leiden, The Netherlands

**Keywords:** fatty acids, terpenoid indole alkaloids, Madagascar periwinkle, cell suspension cultures, jasmonic acid, multivariate data analysis

## Abstract

The stress response after jasmonic acid (JA) treatment was studied in cell suspension cultures of *Catharanthus roseus*. The effect of JA on the primary and secondary metabolism was based on changes in profiles of fatty acids (FA) and terpenoid indole alkaloids (TIA). According to multivariate data analyses (MVDA), three major time events were observed and characterized according to the variations of specific FA and TIA: after 0–30 min of induction FA such as C18:1, C20:0, C22:0 and C24:0 were highly induced by JA; 90–360 min after treatment was characterized by variations of C14:0 and C15:0; and 1440 min after induction JA had the largest effect on both group of metabolites were C18:1, C18:2, C18:3, C16:0, C20:0, C22:0, C24:0, catharanthine, tabersonine-like 1, serpentine, tabersonine and ajmalicine-like had the most significant variations. These results unambiguously demonstrate the profound effect of JA particularly on the accumulation of its own precursor, C18:3 and the accumulation of TIA, which can be considered as late stress response events to JA since they occurred only after 1440 min. These observations show that the early events in the JA response do not involve the *de novo* biosynthesis of neither its own precursor nor TIA, but is due to an already present biochemical system.

## 1. Introduction

Fatty acids (FAs) are the most abundant form of reduced carbon chains available from nature and plant FAs represent a large reservoir of diversity with at least 200 different types that occur mainly in plants. In eukaryotic organisms like plants, they consist almost exclusively of 16 and 18-carbon FAs, being palmitic acid (C16:0) the major saturated FA followed by the unsaturated linolenic (C18:3), linoleic (C18:2) and oleic (C18:1) and these are often referred to as the common FAs [1] (Table 1).

**Table 1:** Common fatty acids with their chemical formula, common names and abbreviations.

Systematic Name	Common Name	Formula
Decanoic acid	Capric acid	C10:0
Dodecanoic acid	Lauric acid	C12:0
Tetradecanoic acid	Myristic acid	C14:0
∆-9-Tetradecanoic acid	Myristoleic acid	C14:1
Pentadecanoic acid	Pentadecanoic acid	C15:0
Hexadecanoic acid	Palmitic acid	C16:0
∆-9-Hexadecanoic acid	Palmitoleic acid	C16:1
Heptadecanoic acid	Heptadecanoic acid	C17:0
Octadecanoic acid	Stearic acid	C18:0
∆-9-Octadecanoic acid	Oleic acid	C18:1n9c
∆-9,12-Octadecadienoic acid	Linoleic acid	C18:2
∆-9,12,15-Octadecatrienoic acid	α-Linolenic acid	C18:3n3
Eicosanoic acid	Arachidic acid	C20:0
Heneicosanoic acid	Heneicosanoic acid	C21:0
Docosanoic acid	Behenic acid	C22:0
Tetracosanoic acid	Lignoceric acid	C24:0

Fatty acids are essential molecules present in all living organisms. They serve as major source of reserve energy by being part of complex lipids and are essential components of cellular membranes [2]. Moreover, they are also key molecules that participate in various biological processes [3]. In plants, composition and turnover of intracellular lipids and FAs are frequently altered during development and are among the first targets of environmental cues [4]. For example, the polyunsaturation of FAs has proven to be correlated to adaptation when plants are challenged with high or low temperatures [5,6] or high salinity [7] and consequently they modulate a variety of responses to biotic and abiotic stresses.

One of the best-characterized FA-derived signal molecules is jasmonic acid (JA) along with its derivatives, collectively known as oxylipins or jasmonates (JAs). They play a role of master switch in many plant responses to biotic and abiotic factors such as wound response after herbivore attack, ultraviolet light or ozone, drought, but they also modulate flower, seed and fruit development, seed germination, pollen viability, anthocyanin accumulation, fruit ripening [8], tuberization in *Solanum,* tendril coiling in *Bryonia* and promotion of leaf senescence [9,10,11]. Jasmonic acid and its methyl ester (MeJA) have also a positive effect on the signal transduction chain leading to the accumulation of highly valued terpenoid indole alkaloids (TIA) in *C. roseus*. Both JAs induce most of the known TIA pathway genes [12], which results in higher levels of TIA in cell suspensions of *C. roseus* [13]. The coordinated expression of biosynthetic genes is mediated through the octadecanoid-responsive *Catharanthus* AP2-domain proteins ORCA2 and ORCA3, which are both induced by JA [14,15].

**Scheme 1:**
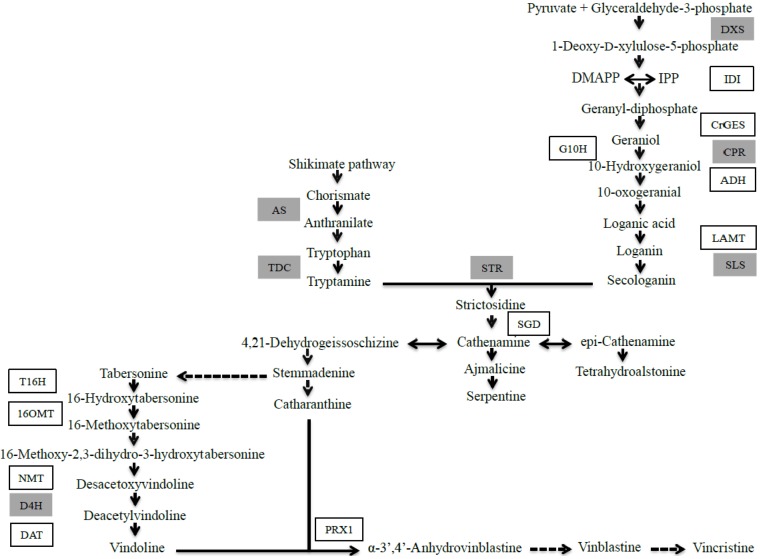
Biosynthetic pathway of TIA in *C. roseus* (modified from [20]). Broken arrows indicate multiple enzymatic reactions. Enzymes with their corresponding cloned gene are indicated. Genes previously reported to be regulated by ORCA3 are indicated in gray boxes.

The accumulation of TIA can be considered as a late response in the jasmonate-mediated stress response in *C. roseus* where the expression of biosynthetic genes such as geraniol 10-hydroxylase (*G10H*), tryptophan decarboxylase (*TDC*), strictosidine synthase (*STR*) and strictosidine β-d-glucosidase (*SGD*) (Scheme 1) takes place after 2 h of induction [12,16] and the accumulation of TIA are detected in significant amounts only after 4–24 h of induction [17,18]. Therefore, since the rapid burst of jasmonates after wounding and feeding with JA takes place within 30 s [19], it is considered as an early response that precedes any transcriptional activity. Further events that involve the *de novo* biosynthesis of precursors, the *de novo* production of enzymes or secondary metabolites is considered as a late event in the JA-mediated stress response. As a part of an integrative study on the fast jasmonate response, we attempted to contribute to the knowledge on FA and TIA accumulation after treating cell suspensions of *C. roseus* with JA to establish a time frame where all these responses occur both in primary and secondary metabolism.

ADH: acyclic monoterpene primary alcohol dehydrogenase; AS: anthranilate synthase; CPR: cytochrome P450 reductase; D4H: desacetoxyvindoline 4-hydrolase; DAT: acetyl-CoA:4-*O*-deacetylvindoline 4-*O*-acetyltransferase; DXS: 1-deoxy-d-xylulose-5-phosphate synthase; G10H: geraniol 10-hydroxylase; CrGES: geraniol synthase; IDI: isopentenyl diphosphate isomerase; LAMT: *S*-adenosyl-l-methionine:loganic acid methyltransferase; NMT: 16-methoxy-2,3-dihydro-3-hydroxytabersonine; 16OMT: 16-hydroxytabersonine-16-*O*-methyltransferase; PRX1: peroxidase 1; SGD: strictosidine β-d-glucosidase; SLS: secologanin synthase; STR: strictosidine synthase; TDC: tryptophan decarboxylase; T16H: tabersonine 16-hydroxylase.

## 2. Results and Discussion

### 2.1. JA Induces the Biosynthesis of Its Own Precursor, C18:3

The most abundant FAs present in suspension cultures of *C. roseus* were C16:0, C18:2 and C18:3 as previously reported in cell systems of *C. roseus* [6,8,21,22,23,24,25] whereas C10:0, C15:0, C21:0 and C24:0 were detected as traces. Even-numbered species were predominant over the odd-numbered ones e.g., C9:0, C11:0, C13:0 and C15:0 [26,27,28]. Very long chain FAs such as C20:0, with a high predominance of even-numbered series, are compounds that rarely occur in suspension cultures, since they are mainly found in waxy surfaces as well as in seed oils [29]. Nevertheless, polyunsaturated FAs with chain lengths up to C24:0 have been reported for calli and cell suspension cultures of different plant species [24,30,31,32,33].

**Figure 1:**
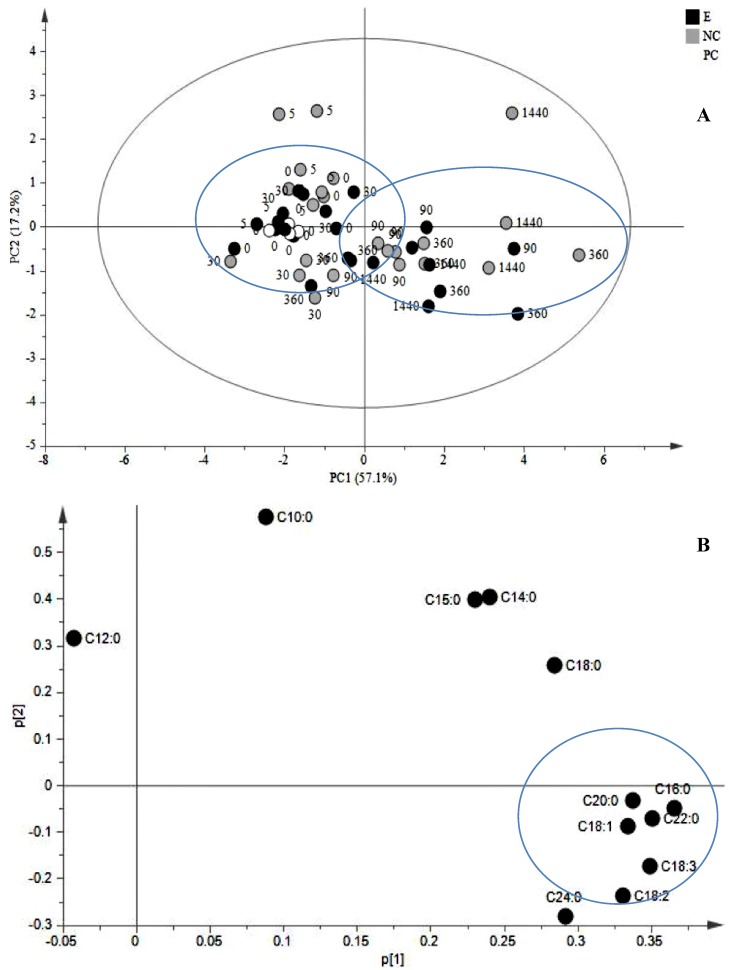
Multivariate data analysis of cell suspensions of *C. roseus* treated with JA. (**A**) PCA scores plot generated from PCA using GC-MS normalized data (PC1 *vs.* PC2) of treated cells and controls. The variances accounted by PC1 and PC2 were 57.1% and 17.2%, respectively. (**B**) PCA loading plot from PCA (PC1 *vs.* PC2) showing the most influential FA. Black circles: JA-treated cells; white circles: untreated cells; grey circles: mock (40% EtOH).

According to the principal component analysis (PCA) scores plot of PC1 *vs.* PC2 (Figure 1A), a clear separation was achieved by PCA score plot where two main groups were observed: 0–5–30 min and 90–360–1440 min after elicitation. Examination of PCA loading plot of JA-treated cells showed that C16:0, C20:0, C18:3, C18:2, C22:0 and C18:1 and C24:0 (Figure 1B) were the most influential FA for the separation with the highest variation in the model.

These observations suggest that JA is able to induce the biosynthesis of its own precursor, that is C18:3, but only at later time points *i.e.*, 1440 min after induction which also means that changes in the FA composition of the membrane are not involved in the fast response after induction with JA.

A variation in FA profile after JA-elicitation suggests a remodeling in the lipid architecture of the membrane where the cell has to cope first with non-physiological amounts of JA with an efficient and fast mechanism of signal attenuation mainly accomplished by hydroxylation [34], epimerization or methyl esterification [35]. In wound-induced tissues, further responses involve the release of precursors from the membrane mediated by lipases to generate the substrates for JA synthesis as occurred for wound-induced leaves of tomato, where 15-fold increase of levels of C18:3 peaked 1 h after wounding, which correlated with an increase in JA [36,37]. These results differ from ours, where levels of C18:3 had significant variations after 1440 min of treatment suggesting that at least in *C. roseus*, the JA-mediated response does not involve the relocation of precursors like C18:3 but the induction of the stress response pathway as it has been demonstrated in previous experiments where the exogenous application of C18:2 and C18:3 as elicitors, leads to the enhanced accumulation of secondary metabolites in root cultures of *Panax ginseng* [38,39], cell suspension cultures of *Lycopersicon esculentum*, *Tinospora cordifolia*, *Erythrina cristagalli* and *Eschscholtzia californica* [40] and LOX activity in *N. tabacum* cell cultures after 1–2 h of induction [41] suggesting the *de novo* biosynthesis of precursors.

**Figure 2:**
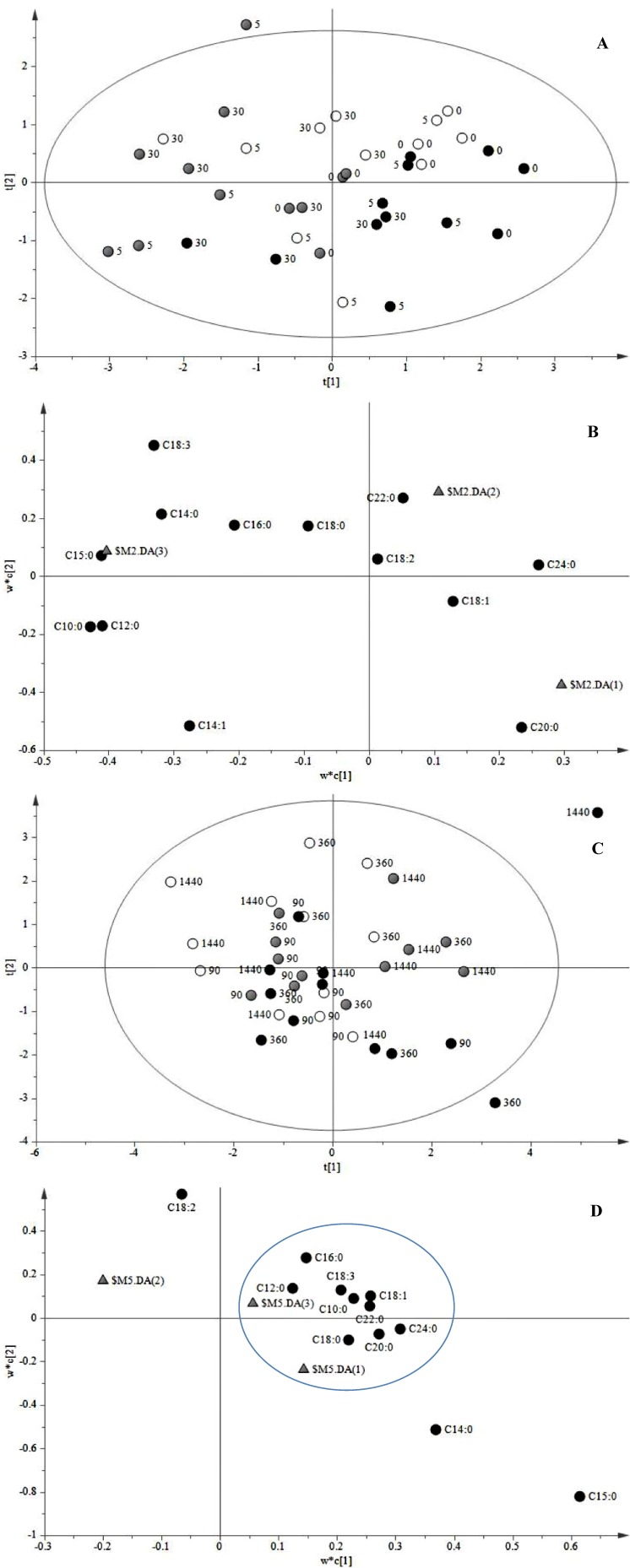
PLS-DA of cell suspension cultures of *C. roseus* treated with JA. (**A**) Scores plot of FA forming the first class (0–5–30 min after induction). (**B**) Loadings plot of the first class (0–5–30 min after induction. (**C**) Scores plot of FA forming the second class (90–360–1440 min after induction). (**D**) Loadings plot of the second class (90–360–1440 min after induction). Black circles: JA-treated cells; white circles: untreated cells; grey circles: mock (40% EtOH).

Further analysis of the influence of JA on the FA profile in cells of *C. roseus* was observed by Partial least squares-discriminant analysis (PLS-DA) of the scores and loadings plots of treated cells showing a clustering of two main groups: 0–5–30 min and 90–360–1440 min (Figure 2). The scores (Figure 2A) and loadings (Figure 2B) plots examination of the first group revealed that JA had a marked influence on few FA such as C18:1, C18:3, C20:0 and C24:0 after 30 min of induction whereas the second group (Figure 2C,D) had the most prominent variations in a larger group with similar responses to JA *i.e.*, C16:0, C18:3, C10:0, C18:1, C22:0, C24:0, C22:0 and C12:0.

#### 2.1.1. Effect of JA on TIA Accumulation in Cell Suspensions of *C. roseus*

Jasmonic acid was applied to cell suspension cultures of *C. roseus* on the 4th day after subculture. Seven peaks were noticed and distinguished only by their different retention times (α-TIA-1-7). According to their absorbance under ultraviolet (UV) light, three maxima were observed at 225, 300, 325 nm, which is characteristic of alkaloids possessing an α-methylene indoline chromophore, constituted by a double bond conjugated with a carbonyl group in the α position of the indoline nitrogen atom, like present in tabersonine. However, the aim of this work was not to identify each trace alkaloid present in our cell system but to get a more general overview of the main TIA accumulated after JA treatment.

**Figure 3:**
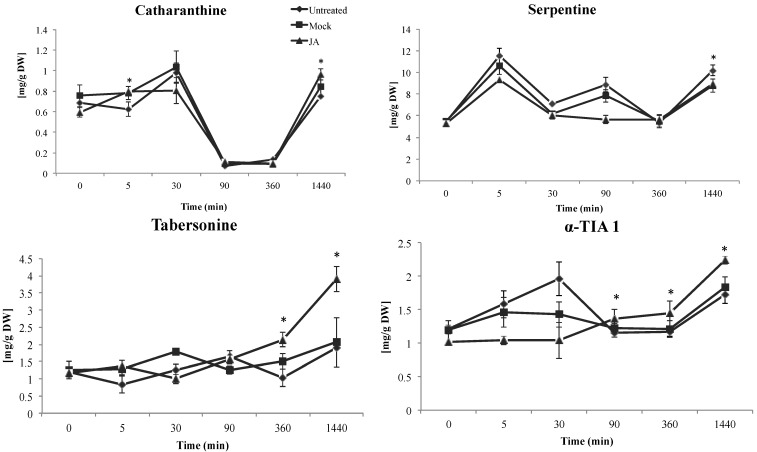
Profiles of the TIA with the most significant variations in cell suspension cultures of *C. roseus* after JA treatment. Values are the mean ± SE of one experiment with four biological triplicates. Significant results are marked with stars (* *p* < 0.05).

The most significant effects were observed on the accumulation of α-TIA-1 and tabersonine after 360 min of induction whereas catharanthine and serpentine were induced after 1440 min of elicitation (Figure 3). Levels of catharanthine, α-TIA-1 and tabersonine transiently increased after 5 and 360 min of elicitation, respectively, reaching a maximum after 1440 min where their accumulation was clearly induced by JA. Previous investigations on cell suspensions of *C. roseus* treated with 100 µM of MeJA, have shown a 300% increase in alkaloid accumulation like ajmalicine and serpentine [14] and strictosidine [42]. This is in contrast with our observations and previous reports on the same cell line [43] where only serpentine but not strictosidine was induced by JA.

Treatment with JA did not induce the accumulation of vindoline in cell suspension cultures of *C. roseus* and consequently that of bisindole alkaloids in agreement with previous knowledge [44]. This inability is mainly due to the lack of expression of *D4H* and the transcriptional inactivation of *DAT* [45,46]. This can be explained with the tight regulation in the biosynthesis of vindoline where the enzymes are located in different cellular compartments. The first step in the conversion from tabersonine to vindoline is its hydroxylation [47]. Regardless the fine-tuned regulation in vindoline biosynthesis, some transcripts specific to its biosynthesis have been detected in hairy roots and cell suspension cultures of *C. roseus* [48]. However, exposure of cell cultures of *C. roseus* to light increased the expression of *T16H* within 22–28 h of treatment [49] and was also found along with *16OMT* to be expressed in cell suspensions cultures in 20% lower levels than in young leaves of *C. roseus* [50]. In addition, activities of T16H and *O*-methyltransferase but not that of NMT, D4H and DAT are present in cell suspensions of *C. roseus* indicating a degree of separation in the regulation of the first two and last four steps in the conversion of tabersonine to vindoline [50].

#### 2.1.2. Late Events in the JA Stress Response

**Figure 4:**
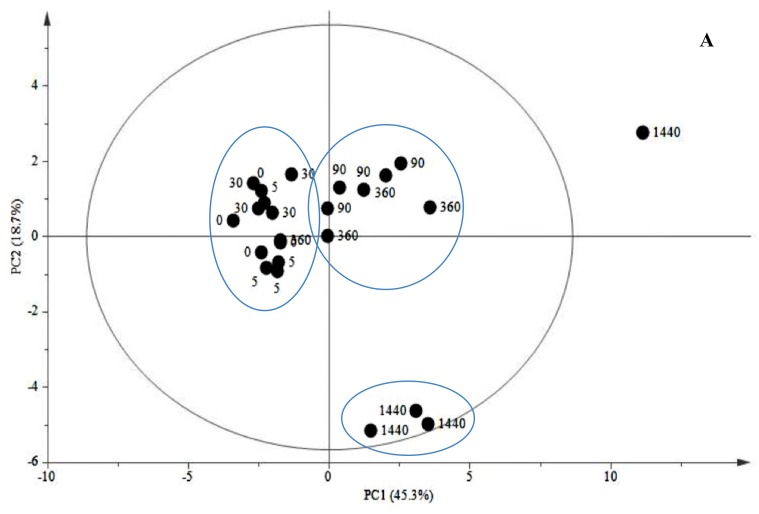

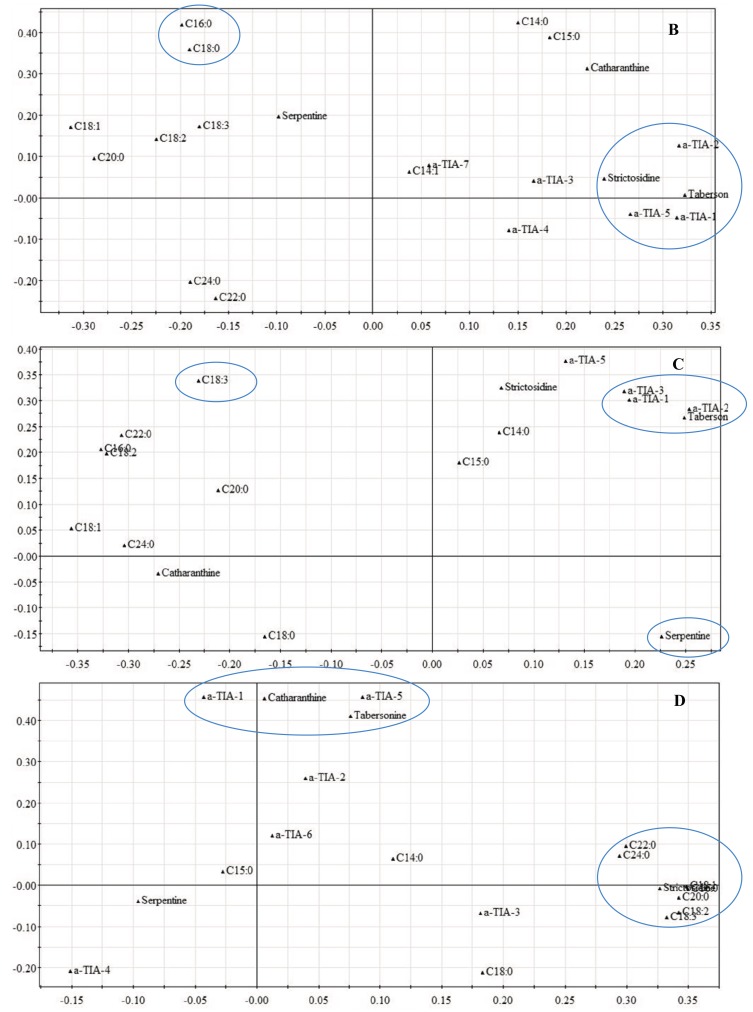
PCA analysis of scores and loadings plots of FA and TIA in cell suspensions of *C. roseus* after JA treatment. (**A**) PCA scores plot of FA and TIA profiles allowed the separation of data into three different clusters of events: first cluster (0–5–30 min after JA treatment); second cluster (90–360 min after JA treatment) and third cluster (1440 min after JA treatment). Variances accounted by PC1 and PC2 were 45.3% and 18.7%, respectively. (**B**) PCA loadings plot of PC1 *vs.* PC2 of FA and TIA profiles in the first cluster. (**C**) PCA loadings plot of the second cluster. (**D**) PCA loadings plot of the third cluster.

Analysis of PCA scores of FA and TIA allowed the classification of three major time events characterized according to the specific pattern of each compound over time. The three major clusters after JA treatment were 0–5–30 min; 90–360 min and 1440 min (Figure 4A). In the first cluster, JA-treated cells were characterized by variations in compounds like C16:0, C18:0, strictosidine, α-TIA-1, α-TIA-2 and α-TIA-5 (Figure 4B). After 90 min of treatment, changes were associated to variations in C18:3, serpentine, catharanthine, α-TIA-1-3 and tabersonine (Figure 4C). The most significant variations were observed after 1440 min of treatment were JA had a stronger effect on TIA accumulation where alkaloids such as tabersonine, α-TIA-1, α-TIA-5 catharanthine and vindoline-like were highly induced by JA and another group also induced by JA was conformed by strictosidine, C18:3, C18:2, C18:2, C20:0, C24:0, C22:0 and C16:0 (Figure 4D).

Jasmonic acid exerts well-documented differential responses both in cell systems and in plants in the stress response. Wound-induced experiments in *Arabidopsis* have shown that the accumulation of JA starts within 120 s after wounding and spreading the signal to long distance unwounded leaves at an average velocity of 3.4–4.5 cm min^−1^ [19]. Induction of JA accumulation in cell suspension cultures of *Rauvolfia canescens* takes place within 20–30 min after exposure to a biotic elicitor, reaching a maximum concentration of the free acid after 90 to 240 min [37,51], which is in complete agreement with our results. These results suggest that *de novo* biosynthesis of JA starting from the release of C18:3 from thylakoids is very unlikely since lipoxygenase 2 (LOX2) is known to contribute to almost 75% of JA measured at 24 h after wounding leaves [52]. Mutants in the LOX pathway such as *lox2-1* and *lox6A*, were still able to accumulate low levels of JA and JA-Ile in the first 5 min upon wounding but caused > 99% reduction in the concentrations of 12-*oxo*-phytodienoic acid (OPDA) and dinor-12-*oxo*-phytodienoic acid (dnOPDA)-containing arabidopsides in wounded leaves [19] and reduction of *JAZ10* expression [53] only indicating the redundancy of this family of enzymes but confirming that the fast response is not mediated by them.

Interestingly, when the ability of the precursor of JA *i.e.*, C18:3 to induce TIA accumulation in hairy roots of *C. roseus* was tested and compared to that of JA, it was found that the octadecanoid pathway is not responsible of the production of TIA [54]. Thus, the effects of feeding JA on TIA production to any system are related to pathway induction and not to the relocation of precursors. In contrast, ajmalicine production is highly inducible by MeJA in cell suspension cultures of *C. roseus* where the highest yields (4.75 mg/L) was observed when cells were treated with 3 mM of CaCl_2_ and 100 µM of MeJA suggesting that cells were dependent on a low intracellular Ca^2+^ concentration [55], which is in contrast with the previous knowledge that Ca^2+^ and MeJA induce the production of the transcriptional factors CrBPF-1 (*Catharanthus roseus* Box P Binding Factor-like protein 1) and ORCAs that regulate the expression of *STR* [56].

The very fast burst of JA accumulation excludes any transcriptional activation assuming that the endogenous JA is formed from a readily and abundantly precursor and released through a constitutively enzymatic process. A likely scenario includes the liberation and conversion of OPDA and/or dnOPDA both esterified to a monogalactosyldiacylglyceride (MGDG) and/or digalactosyldiacylgliceride (DGDG) into JA, a system so far only found in *Arabidopsis* [57,58].

The time line of events including primary and secondary metabolites *e.g.* fatty acids and alkaloids after JA-induction, places their accumulation into a late response since significant increases in C18:3 along with tabersonine, catharanthine, serpentine and α-TIA-1 were observed only after 360 min of JA induction with peaking levels after 1440 min. Based on these observations and previous knowledge where C18:3 does not induce neither JA nor TIA accumulation in hairy roots of *C. roseus*, we support the idea that two independent time-events occur after JA induction where in the early response *i.e.*, after 5–30 min of induction, responses include a rapid propagation and intensification of the signal as an “alert-state” to accumulate enough endogenous amounts of JA to enter into a late response *i.e.* after 6–24 h, where the *de novo* biosynthesis of precursors like C18:3 and the accumulation of TIA are the result of gene expression mediated through the JA-mediated signaling pathway.

## 3. Experimental Section

### 3.1. Cell Cultures and Elicitation with Jasmonic Acid

Cell suspension cultures of *C. roseus* cell line CRPP were grown in 250 mL Erlenmeyer flasks containing 50 mL of Gamborg B5 [59] medium supplemented with 30 g/L sucrose and 1.86 mg/L 1-naphthaleneacetic acid (NAA) and adjusted to pH 5.8 with 0.1 N KOH [43]. Cell cultures were propagated on a rotary shaker (110 rpm) at 25 °C under continuous light (500–1500 lx) and were subcultured every three weeks. Four-day-old cell suspension cultures were treated with 101.9 µM/70 mL of culture medium of JA (Sigma-Aldrich, St. Louis, MO, USA) or 150 µL of 40% v/v EtOH (mock) or nothing (untreated) and were harvested in quadruplicates at 0, 5, 30, 90, 360 and 1440 min after elicitation. Cells were filtered on Whatman filter paper under partial vacuum and biomass and media samples were immediately frozen in liquid nitrogen and kept at −80 °C until further analysis.

### 3.2. Chemicals Used for Cell Suspension Cultures

The chemicals used for macro salts were CaCl_2_ (min. 99%), KH_2_PO_4_ (min. 99.5%), KNO_3_ (min. 99%) and NH_4_NO_3_ (min. 99%) were purchased from Merck (Darmstadt, Germany) and MgSO_4_ was obtained from OPG Farma (BUVA BV, Uitgeest, The Netherlands). The chemicals used for micro salts were H_3_BO_3_, MnSO_4_·H_2_O, ZnSO_4_·7H_2_O, Na_2_EDTA (Merck) and FeSO_4_·7H_2_O (Brocades-ACF Groothandel NV, Maarssen, The Netherlands) were dissolved into one solution and KI, NaMoO_4_·2H_2_O, CuSO_4_·5H_2_O and CoCl_2_·6H_2_O (Merck) were dissolved into another solution to avoid insolubility. Thiamine-di-HCl was from Janssen Chimica (Geel, Belgium), pyridoxine-HCl was from Sigma-Aldrich (St. Louis, MO, USA); nicotinic acid (99.5%) and glycine (99.7%) were purchased from Merck, as was NAA, Sucrose (99.7%) and *myo*-inositol (99.7%) were from Duchefa Biochimie (Haarlem, The Netherlands).

### 3.3. Chemicals Used for Fatty Acid Determination and Alkaloid Standards

The mixture of 37 FA methyl esters (37 Component FAME Mix) was obtained from Supelco (Sigma-Aldrich; Bellefonte, PA, USA). All other chemicals and solvents were of analytical grade and purchased from common sources. Water was treated in a Milli-Q water purification system (TGI Pure Water Systems, Brea, CA, USA). Strictosidine and secologanin were provided by Phytoconsult (Leiden, The Netherlands); loganic acid, tabersonine and vindoline were purchased from PhytoLab (Vestenbergsgreuth, Germany); tryptamine was purchased from Sigma-Aldrich Chemical (Milwaukee, WI, USA) tryptophan and ajmalicine were purchased from Sigma-Aldrich; serpentine was purchased from Roth (Karlsruhe, Germany) and catharanthine were kindly gifted by Pierre Fabre (Gaillac, France).

### 3.4. Fatty Acid Extraction

Samples of 10 mg of freeze-dried cells were spiked with 50 µg of C17:0 as internal standard and then subjected to hydrolysis with 1 mL of 1M of KOH in 95% EtOH. The suspension was ultrasonicated for 10 min followed by heating the closed tube at 80 °C for 30 min. After cooling at room temperature, 1 mL of Milli Q water was added followed by two times extraction with 1 mL of *n*-hexane containing 0.01% butylated hydroxytoluene (BHT; Sigma-Aldrich), after vigorously vortexing, samples were centrifuged for 10 min at 3,500 rpm and the upper hexane layer was removed and discarded. The aqueous layer was acidified with 6 M HCl and extracted twice with 1 mL *n*-hexane (0.01% BHT) and after centrifugation for 10 min at 3,500 rpm upper layers were pooled and completely dried under a gentle flow of N_2_ gas. To the residues 1 mL of boron trifluoride (BF_3_; Sigma-Aldrich) in MeOH was added and the closed tubes were heated for 15 min at 80 °C and then cooled down at room temperature and 1 mL of *n*-hexane (0.01% BHT) was added and after centrifugation, the upper layer (300 µL) was used in GC-MS analysis.

### 3.5. Gas Chromatography-Mass Spectrometry

FAME analysis was performed on an Agilent 7890A series gas chromatograph equipped with an Agilent 7693 auto sampler and an Agilent 5775C Triple-Axis MSD detector (all from Agilent Technologies Inc., Santa Clara, CA, USA) and separated on a 30 m × 0.25 mm I.D. × 0.25 µm film thickness DB-Wax column (J&W; Agilent Technologies Inc.), with a constant flow of 20 mL/min of He as a carrier gas. The injection port was heated to 250 °C. The injection volume was 1 µL with a split ratio of 20:1. The oven temperature was 50 °C for 1 min, then 25 °C/min to 200 °C and then 3 °C/min to 250 °C for 18 min. All mass spectra were acquired in the electron impact (EI) mode for full scan in total ion current (TIC) and selected ion monitoring (SIM) modes. GC-MS was controlled by Enhanced Chemstation software version E.02.00.493 (Agilent Technologies Inc.). Ions selected for quantification are listed in Table 1. The 37 Component FAME Mix was used as a control for possible retention time shifts and mass spectra ion identification.

### 3.6. Alkaloid and Precursor Extraction for HPLC

A modified extraction protocol was followed after [60]. Briefly, freezed-dried samples of 50 mg were extracted twice with 5 mL of MeOH, vortexed for 1 min, sonicated for 20 min and then centrifuged for 30 min at 3,500 rpm. Pooled samples were reduced to dryness under reduced pressure. To the dried extract 500 µL of 1 M H_3_PO_4_ was added and the suspension was thoroughly homogenized and then transferred to an Eppendorf tube for centrifugation for 10 min at 13,000 rpm. Extracts were filtered with a 0.2 µm PTFE membrane and then 50 µL were analyzed by HPLC.

### 3.7. HPLC Analysis

Chromatographic separations were carried out on a 250 mm × 4.60 mm, 5 µm Gemini-NX C18 (Phenomenex Inc., Torrance, CA, USA) column and a guard column filled with RSil C_18_ HL (Zeochem AG, Uetikon, Switzerland) at room temperature. Elutions were performed at a flow rate of 1.5 mL/min. Solvents used for TIA were 80% 5mM Na_2_HPO_4_ pH 7 adjusted with H_3_PO_4_ (Solvent A) and 20% ACN (Solvent B) and for precursors 88% 0.01M H_3_PO_4_ (Solvent A) and 12% ACN (Solvent B). Gradient analyses modified from [61] were as follows: 80:20 (A:B) to 20:80 in 31 min for TIA and 88:12 (A:B) to 30:70 and then to 88:12 in 25 min for precursors, injection volume was 50 µL. The HPLC system was equipped with an Agilent 1200 Series coupled to an Agilent G1315D photodiode-array detector. It consisted on of a G1310A binary pump, a G1329A autosampler, a G1322A degasser and a G1315D photo-diode array detector controlled by ChemStation software (Agilent v. 03.02; all from Agilent Technologies Inc.). Spectroscopic data from all peaks were accumulated in the range of 220–320 nm and chromatograms were recorded at 220, 254, 280, 306 and 320 nm. Detection and quantification was achieved by comparison of their absorbance with that of reference standards.

### 3.8. Data Handling

Differences between treatments, among time points and groups of TIA, regarding contents of each individual TIA in the positive controls were assessed using the Mann–Whitney non-parametric test using R-Project software (v 3.0.0). A *p*-value less than 0.05 was considered statistically significant.

Fatty acids were identified as FAME with the help of the National Institute of Standards and Technology (NIST) library version 2.0f (Agilent Technologies Inc.). Quantification was done by normalizing the peak area of each FA species with that of the internal standard (C17:0). Differences between days and between TIC and SIM, as well as between groups of FA, regarding contents of each individual FA in the positive controls were assessed using the Mann–Whitney non-parametric test using R-Project software (v 3.0.0). A *p*-value less than 0.05 was considered statistically significant. Principal component analysis (PCA) was performed with the SIMCA-P software (v 13.0, Umetrics, Umeå, Sweden). Normalized peak area data was scaled using unit variance (UV). The output from the PCA consisted of a scores plot, which gave an indication of the discrimination of the profile of 13 different FA and a loadings plot, which allowed the identification of those FA with significant influence on the separation of the clustering data.

## 4. Conclusions

Using an MS-based targeted profile, we demonstrate a clear effect of jasmonic acid over time on the FA and TIA accumulation in cell suspensions of *C. roseus* where compounds such as C18:3, C18:2, tabersonine, catharanthine and serpentine had the highest variations. It is very interesting to note that JA applied as an exogenous elicitor induced the accumulation of its own precursor C18:3 but only after 90 min of treatment which cannot explain the fast response seen in the accumulation of JAs. This can only be explained by a larger pool of an intermediate or precursor of JA that is released and immediately activated by a constitutive enzyme system into JA.
